# Comparison of biomechanical behavior between a cast material torso jacket and a polyethylene based jacket

**DOI:** 10.1186/1748-7161-10-S1-O71

**Published:** 2015-01-19

**Authors:** Robert Rizza, XueCheng Liu, John Thometz, Channing J Tassone

**Affiliations:** 1 Dept. of Mechanical Engineering, Milwaukee School of Engineering Milwaukee, WI, USA; 2 Department of Orthopaedic Surgery, Medical College of Wisconsin, WI, USA

## Introduction

Numerous designs are often used in the treatment of early onset of scoliosis. A Thoraco-Lumbo-Sacral Orthosis (TLSO) is constructed using Polyethylene (PE). A series of casting is implemented using cast material. The Cast material is less dense, allows the skin to breathe, and is made of a biodegradable water based resin (3M, BSN Medical). TLSO braces provide correction mechanically through constraint forces, which are minimized with excessive lateral defection.

## Objectives

This study was motivated by goal to compare the biomechanical behavior of the cast based jacket with a PE based design.

## Materials and methods

**Figure 1:**
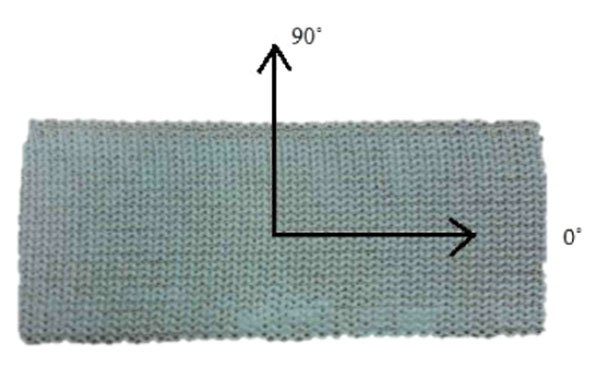


Samples of cast material (Delta-Cast Soft, BSN Medical) were tested for mechanical properties (Young’s Moduli, Poisson ratio and shear modulus). The cast material is a composite material with properties varying in different directions (Figure 1). A finite element model of a patient’s brace was created using an optical scan of the brace. The number of layers in the cast model was varied to determine the number of optimal layers. For the PE, a thickness of 4 mm was used. Loads applied to the brace by the torso were held constant and the lateral defection determined.

## Results

**Figure 2:**
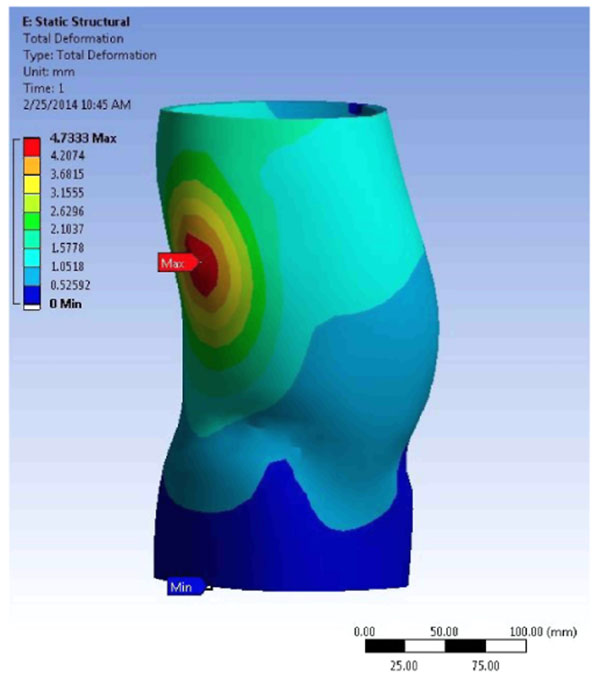


The simulations indicate that the cast jacket with 6 layers will generate at most 4.7 mm deformation. The PE brace will generate a deformation of 2 mm (Figure 2). The structural factor of safety (FOS) for the cast brace was found to be 5.71 and 2.70 for the PE design. The mass of the cast design was 0.175 kg compared to 0.643 kg for the PE design. Material costs for the cast design would be $25.95 and for the PE design $51.90.

## Conclusions

Both designs will generate the proper constraint forces to maintain spinal correction. Based on the design parameters (thickness, mechanical properties, FOS and cost) the brace made of cast material, though slightly thicker (6.6 mm compared to 4 mm) would be 3.5 times lighter and cost half as much (based on material costs). Furthermore the cast brace has double the strength. Thus, from the biomechanical point of view, the cast brace is more efficient than the PE brace.

